# ﻿A new species of *Rhagophthalmus* Motschulsky, 1854 (Coleoptera, Rhagophthalmidae) from Laos represents the smallest known member of the genus

**DOI:** 10.3897/zookeys.1184.112437

**Published:** 2023-11-14

**Authors:** Gabriela Packova, Robin Kundrata

**Affiliations:** 1 Department of Zoology, Faculty of Science, Palacky University, 17. listopadu 50, 77146 Olomouc, Czech Republic Palacky University Olomouc Czech Republic

**Keywords:** Bioluminescent beetles, distribution, diversity, Elateroidea, identification key, neoteny, Oriental Realm, Southeast Asia, taxonomy

## Abstract

*Rhagophthalmus* Motschulsky, 1854 is the most speciose genus in Rhagophthalmidae, distributed in the region encompassing South, East, and Southeast Asia. Here, we describe *R.nanus***sp. nov.** from the Houaphanh Province of eastern Laos, which represents the smallest known species in *Rhagophthalmus* and one of the smallest in Rhagophthalmidae. We compare it with the morphologically similar and geographically close congeners and provide a preliminary identification key to adult males of *Rhagophthalmus* species from mainland Southeast Asia. Additionally, we discuss the morphology and variability of male genitalia within *Rhagophthalmus*.

## ﻿Introduction

The family Rhagophthalmidae (Coleoptera, Elateroidea) includes 66 species classified in 12 genera distributed in South, East, and Southeast Asia (Kundrata et al. 2022). These soft-bodied beetles are characterized by a significant sexual dimorphism. While males are fully winged, all known females are larviform and, similar to predaceous larvae, occur in soil and leaf litter (Wittmer and Ohba 1994; Li et al. 2008; Kawashima et al. 2010). Such modifications in female morphology in various elateroid groups are related to paedomorphosis (e.g., Cicero 1988; Ferreira and Ivie 2022; Hoffmannova and Kundrata 2022). Another interesting phenomenon present in Rhagophthalmidae is the ability to emit light (bioluminescence) which they share with their closest relatives, i.e., Phengodidae, Lampyridae (fireflies), and Sinopyrophoridae (Kusy et al. 2021). No fossils of Rhagophthalmidae are known to date; however, the first fossil of their sister-group family Phengodidae was recently described from the mid-Cretaceous Burmese amber (Roza et al. 2023).

*Rhagophthalmus* Motschulsky, 1854, is the most species-rich genus in the family Rhagophthalmidae. To date, 34 species have been described mainly from East Asia. The highest diversity of this genus lies in China, however several species are distributed in the region ranging from India and Sri Lanka through the Himalayas, to Japan and Indonesia (Kundrata et al. 2022). *Rhagophthalmus* is in need of a complete taxonomic revision. Its generic limits were challenged by some authors, especially considering its relationships with the genera *Ochotyra* Pascoe, 1862 and *Menghuoius* Kawashima, 2000. Although the synonymy of *Ochotyra* with *Rhagophthalmus* (Wittmer and Ohba 1994) is generally accepted, the status of *Menghuoius* differs among authors (Li et al. 2008; Kawashima et al. 2010; Kundrata and Bocak 2011; Liu et al. 2020). Li et al. (2008) synonymized *Menghuoius* with *Rhagophthalmus* based on the similar morphology of their highly paedomorphic larviform females, which, however, bear significantly fewer diagnostic characters than adult males. Kundrata et al. (2022) considered *Menghuoius* a separate genus until a detailed revision of this group is carried out.

During the examination of elateroid material in the collection of the National Museum in Prague, Czech Republic, we identified a yet undescribed *Rhagophthalmus* species from Laos which attracted our attention by its unusually small body size. In this paper, we describe this species as *R.nanus* sp. nov., compare it with its congeners and provide an identification key to adult males of *Rhagophthalmus* species from the mainland Southeast Asia.

## ﻿Material and methods

The genitalia were dissected after a short treatment in hot 10% KOH. Main diagnostic characters were photographed using a digital camera attached to a stereoscopic microscope. The measurements were taken with a scale bar in an eyepiece. Body length was measured from the fore margin of head to the apex of elytra (since abdomen is highly flexible in *Rhagophthalmus*), body/elytra width at humeri, head width including eyes, minimum interocular distance in the frontal part of cranium, maximum eye diameter in the lateral view, pronotal length at midline, scutellar shield length at midline, and scutellar shield at the widest part. We follow the definition of *Rhagophthalmus*, i.e., including *Ochotyra* but excluding *Menghuoius*, by Kundrata et al. (2022). Label data are cited verbatim. The type material is deposited in the National Museum, Prague, Czech Republic (**NMPC**). To compare the new species from Laos with its congeners from adjacent regions, we examined the type and non-type material of *Rhagophthalmus* from the following collections: Muséum national d’Histoire naturelle, Paris, France (**MNHN**), Naturkundemuseum Erfurt, Germany (**NKME**), Natural History Museum, London, United Kingdom (**BMNH**), and the Naturhistorisches Museum, Basel, Switzerland (**NHMB**).

## ﻿Systematics

### ﻿Genus Rhagophthalmus

Taxon classificationAnimaliaColeopteraRhagophthalmidae

Motschulsky, 1854

3444F955-57EC-563B-9B8D-439F962229CD

Rhagophthalmus Motschulsky, 1854: 45. Type species: Rhagophthalmusscutellatus Motschulsky, 1854.
= Ochotyra Pascoe, 1862: 323. Type species: Ochotyrasemiusta Pascoe, 1862: 323 (Wittmer in Wittmer and Ohba 1994: 342).^*^

#### Diagnosis.

This genus can be currently characterized by deeply emarginate eyes, medium-sized mandibles, and relatively short antennae. Its limits and relationships with morphologically similar genera need to be critically revised.

### ﻿ Rhagophthalmus nanus sp. nov.

Taxon classificationAnimaliaColeopteraRhagophthalmidae

927A4F3F-DEF6-5C45-BF53-F0E3EB944A87

https://zoobank.org/A9637E03-20B4-415F-A05B-34AC21E1D330

Figs 1 , 2 , 3

#### Type material.

***Holotype***, male, “Laos-NE, Houa Phan prov., 20°13'09–19"N, 103°59'54"–104°00'03”E, 1480–1510 m, Phou Pane Mt., 22.iv.–14.v.2008, Vít. Kubáň leg.” (NMPC).

#### Type locality.

Laos: Houaphanh [Houa Phan] prov., Phou Pan-Gnai [Phou Pane] Mountain.

#### Diagnosis and comparison.

*Rhagophthalmusnanus* sp. nov. represents by far the smallest known species in *Rhagophthalmus* and can be easily recognized based on the following combination of characters: body small (3.50 mm), elongate, 3.50 times as long as wide; pronotum darker than elytra; elytra elongate, both combined 2.55 times as long as wide and 4.55 times as long as pronotum; aedeagus with parameres slightly longer than median lobe, and with phallobase about 1.35 times as long as wide, ventrally partly covering parameres, and with its anterior margin medially deeply emarginate. Two relatively small *Rhagophthalmus* species in the region, i.e., *R.minutus* Kawashima & Satô, 2001 (Thailand) and *R.tonkineus* Fairmaire, 1889 (Vietnam, China, ?Laos), are still larger than *R.nanus* sp. nov., with the body length of 5.80 mm or more. Additionally, *R.minutus* is much darker, having the pronotum blackish and elytra dark brown (pronotum generally reddish dark brown and elytra light brown to brown in *R.nanus* sp. nov.), and the aedeagus with a median lobe distinctly longer than parameres and a phallobase relatively shorter, about 1.20 times as long as wide, not covering the significant portion of parameres in ventral view, and with its anterior margin rounded. *Rhagophthalmustonkineus* has both the pronotum and elytra of roughly similar dark brown coloration, and the median lobe of the aedeagus distinctly longer than parameres. Another species from Laos, *R.laosensis* Pic, 1917, is much larger (9.00 mm), has relatively shorter and wider elytra (about twice as long as wide), and the median lobe of aedeagus distinctly longer than parameres.

#### Description.

**Figure 1.**
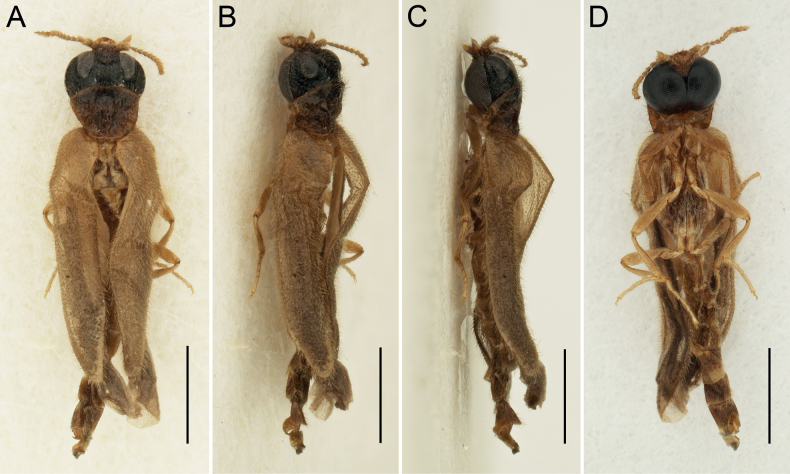
*Rhagophthalmusnanus* sp. nov., holotype, male **A** habitus, dorsal view **B** habitus, dorsolateral view **C** habitus, lateral view **D** habitus, ventral view. Scale bars: 1.0 mm (**A–D**).

***Body*** (Fig. 1A–D) 3.50 times as long as wide (3.50 mm long, 1.00 mm wide at humeri); light brown to brown, with head mostly black; pronotum medially reddish dark brown to blackish, with edges lighter, reddish brown; body surface covered with short light brown setae.

**Figure 2.**
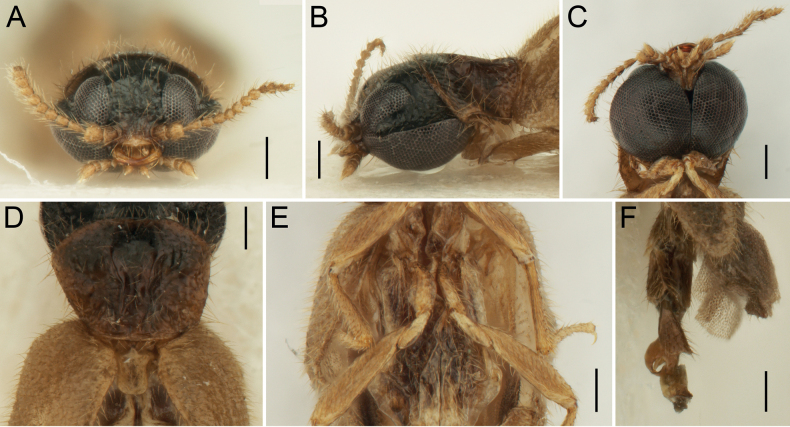
*Rhagophthalmusnanus* sp. nov., holotype, male **A** head, frontal view **B** head, lateral view **C** head, ventral view **D** pronotum, dorsal view **E** thorax, ventral view **F** abdomen, lateral view. Scale bars: 0.2 mm (**A–F**).

***Head*** (Fig. 2A–C) including eyes 1.25 times as wide as pronotum, with short semi-erect setae; area between eyes with surface smooth, shallowly depressed, very sparsely and finely punctate; area in between upper and lower portion of eye sparsely and coarsely punctate. Eyes large, their frontal distance 0.40 times eye diameter, laterally divided into a smaller upper portion and a larger lower portion (Fig. 2B); eyes ventrally contiguous (Fig. 2C). Mouthparts rather small, labrum transverse, partially membranous. Mandibles rather small, narrow, unidentate, sickle-shaped, strongly curved; basally covered with long setae, apical part bare. Maxillary palpi short, 4-segmented, basal palpomere shorter than wide, transverse, palpomere 2 slightly longer than wide; palpomere 3 short, distinctly transverse; ultimate palpomere longest, about twice as long as wide, apically pointed. Labial palpus minute, 3-segmented; palpomere 2 transverse, shorter than basal palpomere; ultimate palpomere similar in shape to ultimate maxillary palpomere, longer than wide, apically pointed. Antenna (Fig. 2A–C) minute, short, surpassing anterior margin of pronotum (when positioned backwards), very slightly serrate, with 12 antennomeres (right antenna without ultimate antennomere); scape and pedicel robust, each slightly longer than wide, pedicel slightly longer than scape; flagellum (antennomeres 3–12) distinctly narrower than scape and pedicel; antennomere 3 longest, distinctly elongate, gradually widened toward apex; antennomeres 4–11 relatively short and subequal in length; antennomere 12 longer than wide, distinctly narrowed apically.

***Pronotum*** (Fig. 2D) transverse (0.55 mm long, 0.70 mm wide), about 1.30 times wider than long when width measured anteriorly (pronotum is widest posteriorly but since both posterolateral portions of pronotum are shriveled, the real shape of pronotum cannot be observed), slightly convex, widest at about posterior angles (pronotum shrunken in dry condition, giving the false appearance that it is widest anteriorly). Anterior margin widely rounded; lateral margins slightly rounded, posterior margin almost straight. Anterior angles inconspicuous; posterior angles short, apically rounded. Lateral pronotal carina complete. Surface of disc relatively smooth, sparsely covered with fine punctures and semi-erect setae, which are slightly longer than those on head and elytra. Prosternum distinctly transverse, with prosternal process reduced to short median protrusion. Scutellar shield (Fig. 2D) tongue-shaped, almost twice as long as wide; sides shallowly and widely emarginate, apex widely rounded; sparsely covered with fine punctures and short semi-erect setae. Mesoventrite short, with median depression. Metaventrite with distinct long discrimen. Elytra (Fig. 1A–C) elongate, subparallel-sided (2.55 mm long, 1.00 mm wide at humeri), both combined 2.55 times as long as wide, 4.55 times as long as pronotum, with apices strengthened, separately rounded, surface rather rough and sparsely covered with short, sub-erect setae. Leg (Fig. 1D) slender; coxa elongate; femur elongate, flattened, obliquely attached to trochanter, distinctly wider than tibia; tibia shorter than femur, gradually slightly narrowed towards apex, apical tibial spurs short and thin; tarsus slender, simple, relatively long, subequal in length to tibia; pro- and mesotarsomeres 1 and 2 subequal in length, metatarsomere 1 longer than metatarsomere 2; in all legs tarsomere 3 shorter than respective tarsomere 2, tarsomere 4 shortest and tarsomere 5 distinctly longest; claw simple, slightly curved.

**Figure 3.**
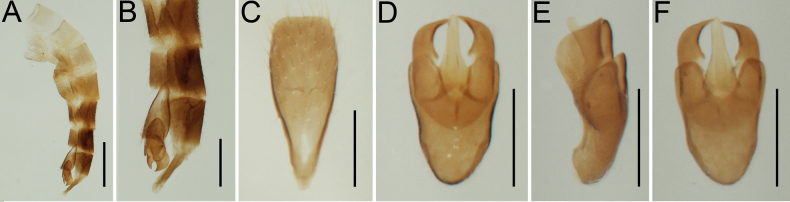
*Rhagophthalmusnanus* sp. nov., holotype, male **A** abdomen, lateral view **B** apex of abdomen, lateral view **C** sternite IX, dorsal view **D** male genitalia, dorsal view **E** male genitalia, lateral view **F** male genitalia, ventral view. Scale bars: 2.0 mm (**A**); 0.5 mm (**B**); 0.2 mm (**C–F**).

***Abdomen*** (Figs 2F, 3A, B) soft, tergites and sternites connected with extensive membranes, very sparsely and finely punctate, with sparse short setae, all tergites except the last one and all ventrites except the last one about subequal in length; tergite X (Fig. 3A, B) free, elongate, narrowed, conical; sternite IX (i.e., last ventrite; Fig. 3C) elongate, about 2.5 times as long as wide, basally narrowed, apically widened and very slightly emarginate. Male genitalia (Fig. 3D–F) about 1.80 times as long as maximum phallobase width; median lobe relatively short, 2.50 times as its maximum width at base, widest at base, abruptly narrowed near middle, then subparallel-sided towards apex, apically rounded, basal struts relatively long, slender and divergent; parameres slightly longer than median lobe, curved, basally joint, medially with short tooth, apically narrowed and sharp; phallobase U-shaped, partly covering parameres in ventral view, about 1.35 times as long as wide and about 1.40 times as long as median lobe, anterior part medially gradually and relatively deeply emarginate, posteriorly slightly narrowed and rounded.

Female and immature stages unknown.

#### Etymology.

The specific epithet “nanus” refers to the small size of this species.

#### Distribution.

Laos (Houaphanh Province).

### ﻿Identification key to the males of *Rhagophthalmus* from mainland Southeast Asia

**Table:** 

1	Pronotum distinctly darker than elytra, reddish dark brown to blackish; elytra yellowish to light brown	**2**
–	Pronotum and elytra more or less uniformly dark brown to blackish	**4**
2	Body length 3.5 mm	***R.nanus* sp. nov.**
–	Body length ≥ 8.0 mm	**3**
3	Parameres narrow; phallobase U-shaped, distinctly longer than wide, covering substantial portion of parameres in ventral view	***R.burmensis* Wittmer in Wittmer & Ohba, 1994**
–	Parameres relatively wide; phallobase more or less transverse, only slightly covering parameres in ventral view	*R.flavus* Kawashima & Satô, 2001/ *R.laosensis* Pic, 1917 ^*^
4	Body length 8.0 mm; antennae dark brown	***R.obscurus* (Pic, 1917)**
–	Body length ≤ 6.6 mm; antennae yellowish brown to brown	**5**
5	Parameres widened towards apex; phallobase basally rounded	***R.tonkineus* Fairmaire, 1889**
–	Parameres narrowed towards apex; phallobase basally truncate	***R.minutus* Kawashima & Satô, 2001**

## ﻿Discussion

The discovery of *R.nanus* sp. nov. from Laos contributes to our better understanding of this little known group of Asian bioluminescent beetles. The species currently classified in *Rhagophthalmus* are usually medium-sized, most of them being 8.0–11.0 mm long. The exception is *R.longipennis* Pic, 1925 from China, which is with 18.0 mm by far the largest species within this genus (Pic 1925). Until the discovery of here described *R.nanus* sp. nov., the smallest representatives of the genus *Rhagophthalmus* were *R.filiformis* Olivier, 1912 from Sri Lanka (5.0–6.0 mm; Olivier 1912) and *R.minutus* from Thailand (5.8 mm; Kawashima and Satô 2001). With the body length of 3.50 mm, *R.nanus* sp. nov. is much smaller than those species and represents not only the smallest known species in the genus *Rhagophthalmus*, but also one of the smallest representatives of the whole family Rhagophthalmidae. Among the formally described rhagophthalmids, the genus *Falsophrixothrix* Pic, 1937 contains small species, ranging from about 4.00 to 6.50 mm. However, this also includes the abdomen since elytra are shortened in this genus. Some undescribed rhagophthalmids are even smaller, having less than 3.5 mm (personal observations of authors).

Li et al. (2008) discussed the importance of male genitalia in distinguishing species of *Rhagophthalmus*. It should also be noted that the authors correctly mentioned that Wittmer and Ohba (1994), followed by some other authors, erroneously mixed up dorsal and ventral sides of the aedeagus. Based on the shape of the aedeagus (information available to authors for 30 out of 35 species), *Rhagophthalmus* can be divided into two separate groups. The first group (at least 19 species) is characterized by the phallobase, which is typically elongate, anteriorly medially emarginate, and covers the substantial portion of parameres in ventral view (e.g., Fig. 3D–F). The parameres are usually surpassing the apex of a median lobe [in for example, the type species *R.scutellatus* or *R.obscurus* (Pic, 1917)]; however, in rare cases the median lobe is slightly surpassing the parameres, as in for example, *R.burmensis* (Wittmer and Ohba 1994) and *R.nanus* sp. nov. The second group (at least 11 species) includes representatives with much smaller phallobase, which is of variable shape (usually wider than long) and never covers the significant portion of parameres in ventral view, and with a median lobe always distinctly surpassing apices of parameres [e.g., *R.flavus*, *R.laosensis*, *R.minutus*, and *R.tonkineus*; see Kawashima and Satô (2001)]. The genus *Menghuoius*, which is morphologically similar to *Rhagophthalmus* and considered its synonym by some authors (Li et al. 2008), has a somewhat intermediate aedeagus, with the more or less transverse phallobase covering only relatively small portions of parameres in ventral view, and a relatively short median lobe with its apex not, or only very slightly, surpassing apices of parameres (Kawashima 2000; Kawashima 2002; Li et al. 2008).

Taking into account above-mentioned morphological differences in male genitalia within *Rhagophthalmus*, it would be interesting to test the phylogenetic relationships among the species of this genus using independent molecular data. However, future phylogenetic studies should focus not only on the relationships within the genus, but also on testing the limits of *Rhagophthalmus* by including morphologically similar genera like *Menghuoius* and *Dioptoma* Pascoe, 1860.

## Supplementary Material

XML Treatment for Rhagophthalmus

XML Treatment for Rhagophthalmus nanus

## Footnotes

For more information, see Kundrata et al. (2022).

Based on the original description of R. flavus and examination of syntype of R. laosensis in MNHN we are unable to reliably distinguish these species.

## ﻿Additional information

### Author ORCIDs

Gabriela Packova https://orcid.org/0000-0001-7949-619X

Robin Kundrata https://orcid.org/0000-0001-9397-1030

### Data availability

All of the data that support the findings of this study are available in the main text.
